# TSMDA: Target and symptom-based computational model for miRNA-disease-association prediction

**DOI:** 10.1016/j.omtn.2021.08.016

**Published:** 2021-08-26

**Authors:** Korawich Uthayopas, Alex G.C. de Sá, Azadeh Alavi, Douglas E.V. Pires, David B. Ascher

**Affiliations:** 1 Structural Biology and Bioinformatics, Department of Biochemistry, University of Melbourne, Parkville 3052, VIC, Australia; 2 Systems and Computational Biology, Bio21 Institute, University of Melbourne, Parkville 3052, VIC, Australia; 3 Computational Biology and Clinical Informatics, Baker Heart and Diabetes Institute, Melbourne 3004, VIC, Australia; 4 Baker Department of Cardiometabolic Health, Melbourne Medical School, University of Melbourne, Parkville 3010, VIC, Australia; 5 School of Computing and Information Systems, University of Melbourne, Parkville 3052, VIC, Australia; 6 Department of Biochemistry, University of Cambridge, 80 Tennis Ct Rd, Cambridge CB2 1GA, UK

**Keywords:** microRNA, disease, miRNA-disease association prediction, target-based similarity, symptom-based similarity, cancer, miRNA-target interaction, XGBoost, machine learning

## Abstract

The emergence of high-throughput sequencing techniques has revealed a primary role of microRNAs (miRNAs) in a wide range of diseases, including cancers and neurodegenerative disorders. Understanding novel relationships between miRNAs and diseases can potentially unveil complex pathogenesis mechanisms, leading to effective diagnosis and treatment. The investigation of novel miRNA-disease associations, however, is currently costly and time consuming. Over the years, several computational models have been proposed to prioritize potential miRNA-disease associations, but with limited usability or predictive capability. In order to fill this gap, we introduce TSMDA, a novel machine-learning method that leverages target and symptom information and negative sample selection to predict miRNA-disease association. TSMDA significantly outperforms similar methods, achieving an area under the receiver operating characteristic (ROC) curve (AUC) of 0.989 and 0.982 under 5-fold cross-validation and blind test, respectively. We also demonstrate the capability of the method to uncover potential miRNA-disease associations in breast, prostate, and lung cancers, as case studies. We believe TSMDA will be an invaluable tool for the community to explore and prioritize potentially new miRNA-disease associations for further experimental characterization. The method was made available as a freely accessible and user-friendly web interface at http://biosig.unimelb.edu.au/tsmda/.

## Graphical abstract

**Figure:**
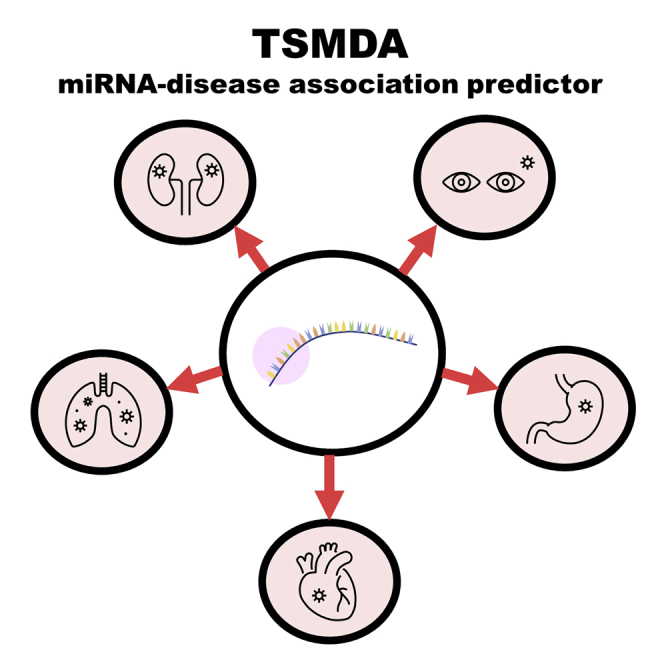

## Teaser

This work proposes a novel model, TSMDA, for predicting miRNA-disease association using miRNA-target and disease-symptom information. TSMDA also encompasses two reliable negative sample selections to effectively predict miRNA-disease associations. TSMDA is freely available as a user-friendly web server for the community to explore potential associations for further experimental miRNA characterization.

## Introduction

MicroRNAs (miRNAs) are small regulatory non-coding RNAs with a typical length of 21–25 nucleotides. Human mature miRNAs control the gene expression of target messenger RNAs (mRNAs) by partially complementary base pairing with the 3′ untranslated region.^1^ This interaction generally results in post-transcriptional repression, occasionally leading to miRNA degradation.^2^ Various physiological processes, such as cell proliferation and cell death, are regulated by a complex network of miRNAs.^2^

The advent of high-throughput sequencing techniques has been contributing to the growing evidence of associations between miRNAs and diseases. Deregulation of several miRNAs is correlated with the development of multiple diseases, such as cancers and brain and cardiovascular diseases.^3^, ^4^, ^5^ For example, pancreatic carcinogenesis may occur from the upregulation of miR-21, miR-155, miR-181, miR-221, and miR-222.^6^ Hence, understanding the relationship between miRNAs and diseases might shed light on pathogenesis, promoting miRNA-based applications such as biomarkers or drugs.^7^, ^8^, ^9^ Currently, a significant number of disease-related miRNAs are experimentally confirmed and collected in multiple databases.^10^, ^11^, ^12^ Despite these significant efforts, large-scale exploration of the potential disease-miRNA associations is unfeasible, since experimental validation is laborious and costly. In this context, effective computational methods are urgently needed to suggest potential associations and guide experimental efforts.

Diverse machine-learning models have been extensively implemented to assist in exploring miRNA-disease relationships.^13^, ^14^, ^15^, ^16^, ^17^, ^18^, ^19^, ^20^, ^21^, ^22^ From the widely accepted assumption that phenotypically similar diseases and functionally equivalent miRNAs tend to be associated, experimentally confirmed associations can be used to identify novel associations. One model in particular, miRNA target-dysregulated network (MTDN), has been built to unveil potential cancer-related miRNAs.^13^ One of the posterior advances is the random forest for miRNA-disease association (RFMDA),^14^ which is based on miRNA functional similarity (MISIM)^23^ and disease semantic similarity,^23^^,^^24^ as features to perform the miRNA-disease-association predictions.

Despite the remarkable effort of currently available methods, model performance was still limited by miRNA and disease similarity estimations that did not directly reflect miRNA mechanisms and disease pathogenesis. The performance improvement obtained by two additional methods, latent feature extraction for miRNA-disease association (LFEMDA)^15^ and distance-based sequence similarity for miRNA-disease association (DBMDA),^16^ emphasize that the introduction of biological features, such as miRNA sequence, into similarity calculation is important. A lack of actual negative samples was also a significant challenge, where various methods randomly selected negative samples from miRNA-disease pairs without confirmed associations.^14^^,^^16^^,^^21^ This approach likely leads to false negatives. Two previous models, non-negative samples extraction (NSEMDA)^17^ and negative sample selection strategy and multi-layer perceptron (NMLPMDA),^18^ have proposed alternative approaches to select reliable negative samples. NSEMDA iteratively filtered unknown samples with positive-unlabeled (PU) learning, an algorithm designed to deal with a labeling issue, where only a single class is available.^25^^,^^26^ Alternatively, NMLPMDA utilized the miRNA-gene-disease network to remove likely associations.^18^

Here we propose a novel machine-learning model that employs target- and symptom-based similarity for miRNA-disease-association prediction (TSMDA). In this study, miRNA target genes and disease symptoms were introduced to enhance similarity calculation, coupled with reliable negative sample selections based on extended miRNA-gene-disease network and modified PU learning.

## Results

### Feature selection

In this study, two feature selection methods, a correlation-based and forward stepwise greedy feature selection,^27^^,^^28^ were employed to select the minimal effective subset from 1,373 features to train a highly accurate model. As a result, 13 features were chosen. This subset consists of five miRNA functional similarities, three target-based miRNA similarities, and five symptom-based disease similarities (Table 1). It is adopted to train and validate the extreme gradient boosting (XGBoost) model.^29^

**Table 1:** Selected features and corresponding biological meaning

Feature	Category	Meaning
1	miRNA functional similarity (MISIM)	similarity with “hsa-miR-1180-3p”
2	miRNA functional similarity (MISIM)	similarity with “has-miR-3179”
3	miRNA functional similarity (MISIM)	similarity with “hsa-miR-320c”
4	miRNA functional similarity (MISIM)	similarity with “hsa-miR-376b-3p”
5	miRNA functional similarity (MISIM)	similarity with “hsa-miR-487a-3p”
6	target-based miRNA similarity	similarity with “hsa-miR-127-3p”
7	target-based miRNA similarity	similarity with “hsa-miR-184”
8	target-based miRNA similarity	similarity with “hsa-miR-516a-5p”
9	symptom-based disease similarity	similarity with “Alopecia (D000505)”
10	symptom-based disease similarity	similarity with “Biliary Atresia (D001656)”
11	symptom-based disease similarity	similarity with “Atopic dermatitis (D003876)”
12	symptom-based disease similarity	similarity with “Myelodysplastic Syndromes (D009190)”
13	symptom-based disease similarity	similarity with “Tourette Syndrome (D005879)”

### Interpretation of the XGBoost model

Model interpretability is one of the essential aspects to consider before putting a machine learning model to use.^30^, ^31^, ^32^ It is crucial for explaining the accuracy of model prediction and guiding performance improvement. Despite achieving high accuracy, popular complex models, such as XGBoost and neural networks,^29^, ^30^, ^31^, ^32^, ^33^ are excessively complex for human interpretation. Different methods have been introduced to help understand the predictions in response to a lack of interpretability.^30^, ^31^, ^32^ SHapley Additive exPlanations (SHAP) is one of the methods designed to explain a model by examining the contribution of each feature in terms of SHAP value to a prediction.^30^ SHAP value is a measure of feature importance, calculated to exhibit the distribution of each feature’s impact on a prediction. The benefits of SHAP values are computational efficiency and consistency with human explanations.^30^

**Figure 1:**
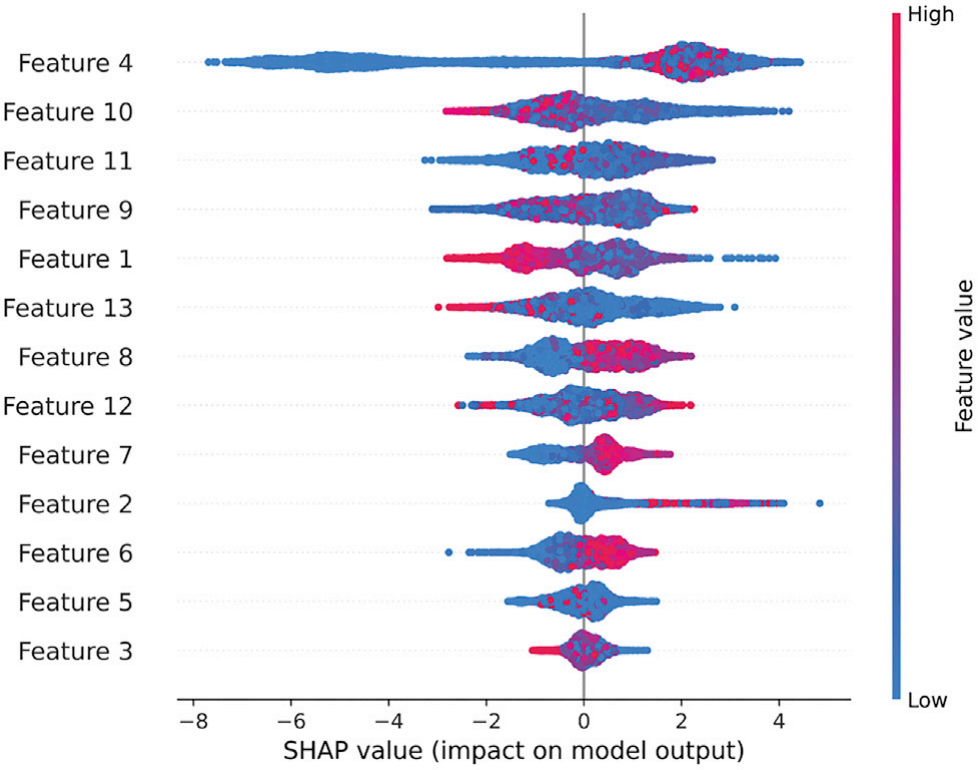
Feature 4 is the most contributing feature to a prediction, showing a distinct positive correlation with a miRNA-disease association The SHAP value for each feature in the XGBoost model was calculated. The features are ranked based on the average impact on a model prediction. One dot represents one miRNA-disease association. The values of features are represented by color, red indicating high values and blue indicating low values.

In this work, we implemented SHAP to analyze how the trained XGBoost model makes a prediction. SHAP values of 13 selected features were calculated and displayed in Figure 1, where features are ranked based on the average impact on model output in descending order. The most important feature is feature 4, representing the MISIM functional similarity with hsa-miR-376b. This miRNA is experimentally supported to be associated with a wide type of diseases, including adrenocortical carcinoma,^34^ cerebral ischemia,^35^ Graves’ disease,^36^ myocardial ischemia,^37^ Parkinson’s disease,^38^ and prostate neoplasms.^39^ According to a widely accepted assumption that similar miRNAs tend to be associated with phenotypically similar diseases, miRNAs with high feature 4 values will be more likely to be associated with these diseases or related conditions. This assumption is in accord with a remarkable positive correlation between feature 4 values and miRNA-disease associations in the figure. Similar trends can be clearly observed in features 6, 7, and 8 that represent target-based miRNA similarity.

Features 10, 11, and 9 are the 2nd, 3rd, and 4th most critical features, accounting for symptom-based disease similarities with biliary atresia, atopic dermatitis, and alopecia. In this case, they present an unclear correlation with miRNA-disease associations. This finding well accords with expectations, as many disease similarities are needed to be considered as a group to represent a particular disease.

### Performance of TSMDA

We started by assessing the ability of TSMDA to predict miRNA-disease associations using The Human microRNA Disease Database (HMDD) v.2.0 database,^10^ assessed under different cross-validation schemes. Under 5-fold cross-validation, our model achieved an AUC of 0.989, as well as Matthews correlation coefficient (MCC), balanced accuracy (bACC), and F1 scores of 0.978, 0.989, and 0.989, respectively (Table 2). The method obtained comparable outcomes from 10-fold and 20-fold cross-validation, further demonstrating the robustness of the TSMDA predictive model (Table 2). Taking a closer look at misclassified entries in a blind test and cross-validation, we noticed that the majority are false negatives. The investigation exhibits that 27 out of 31 entries in the blind test are false negatives. However, no particular miRNA or disease is found predominantly. We further examined the contribution of each feature to misclassified predictions in a blind test with individual SHAP values (Table S1). Unsurprisingly, the result suggested the features with high feature importance, especially feature 4, tend to be the main contributors to a misclassification.

**Table 2:** The results of TSMDA based on a blind test, 5-fold, 10-fold, and 20-fold cross-validation in HMDD v.2.0

Methods	AUC	MCC	bACC	F1
Blind test	0.982	0.965	0.982	0.982
5-fold cross-validation	0.989 ± 0.003	0.978 ± 0.005	0.989 ± 0.003	0.989 ± 0.003
10-fold cross-validation	0.989 ± 0.004	0.978 ± 0.008	0.989 ± 0.004	0.989 ± 0.004
20-fold cross-validation	0.989 ± 0.005	0.978 ± 0.010	0.989 ± 0.005	0.989 ± 0.005

**Figure 2:**
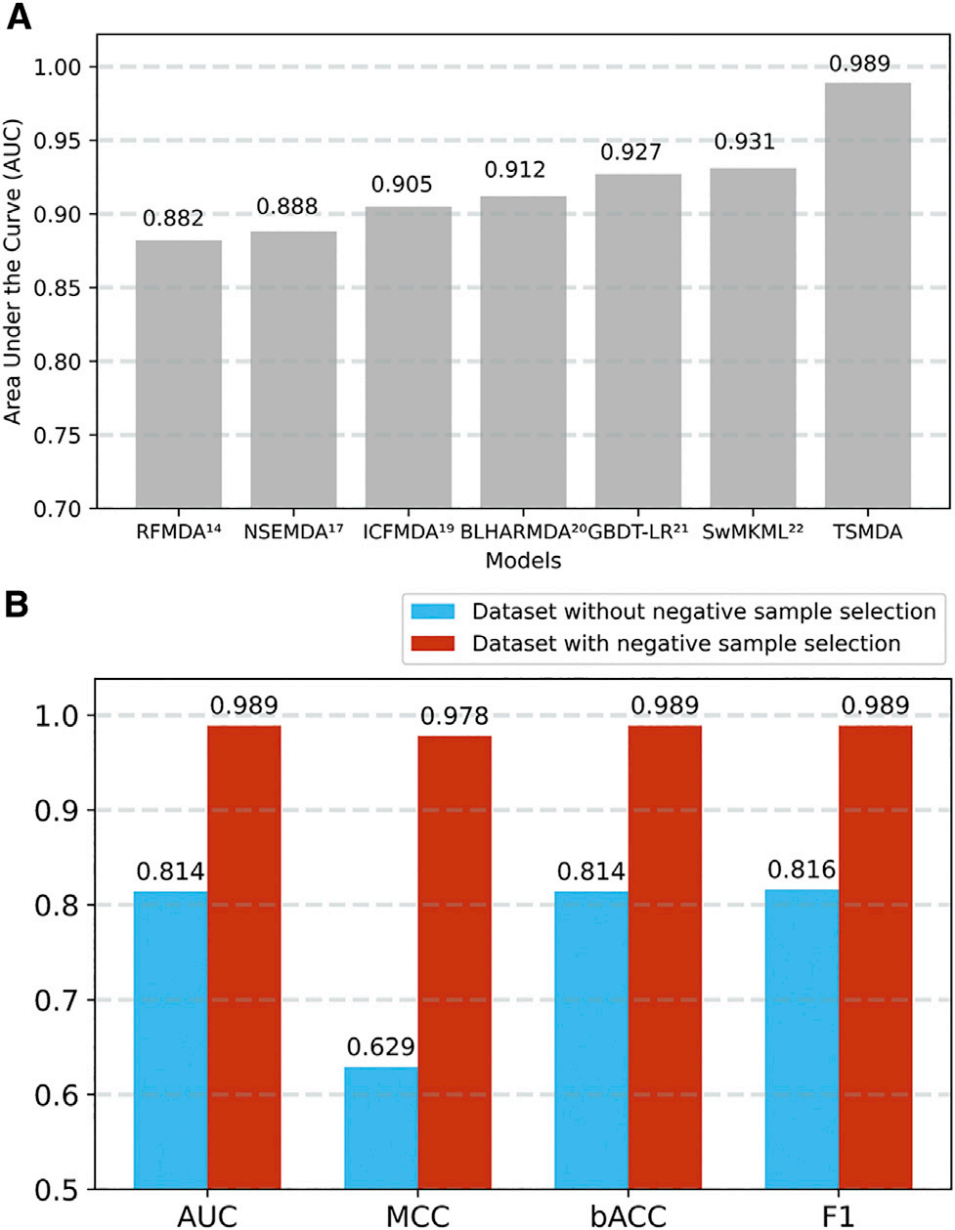
Predictive performance of TSMDA (A) TSMDA considerably outperformed six recent miRNA-disease-association predictive models in terms of area under the curve (AUC). (B) Two negative sample selections, a miRNA-gene-disease network and modified PU learning, substantially enhance the performance of TSMDA. AUC, Matthews correlation coefficient (MCC), balanced accuracy (bACC), and F1 of TSMDA model with and without negative sample were assessed in 5-fold cross-validation with an extreme gradient boosting (XGBoost) classifier.

Diverse computational models have been proposed to fill the missing knowledge of miRNA-disease relationships during the past 10 years.^13^, ^14^, ^15^, ^16^, ^17^, ^18^, ^19^, ^20^, ^21^, ^22^ In this study, we compare the performance of TSMDA with six recent miRNA-disease-association predictors: RFMDA,^14^ NSEMDA,^17^ ICFMDA,^19^ BLHARMDA,^20^ GBDT-LR,^21^ and SwMKML.^22^ The selected methods are based on the same dataset, HMDD v.2.0, enabling an adequate comparison. As most methods are not publicly available for replication, only the AUC values reported in the original article were used for a comparison. As a result, our model considerably outperformed all six recent predictive models (Figure 2A).

We believe one of the reasons behind the performance of TSMDA lies in the novel procedure to measure miRNA and disease similarity by considering target genes and symptoms, which directly reflect the biological nature of miRNAs and diseases. Moreover, unlike previous research that randomly selected negative samples from unknown associations,^14^^,^^16^^,^^21^ TSMDA utilizes a miRNA-gene-disease network, followed by a modified PU learning, to construct more reliable negative samples (Figure 2B).

### Blind test

To evaluate the generalization capabilities of TSMDA, we assessed its performance on an independent blind test of experimentally validated miRNA-disease associations from HMDD, providing an unbiased evaluation of the trained model. The model reached an AUC, MCC, bACC, and F1 of 0.982, 0.965, 0.982, and 0.982, respectively, which were consistent with the performance obtained under cross-validation (Table 2).

### Predicting miRNA-disease associations in cancer

Three case studies involving prevalent cancer types (breast, prostate, and lung cancer) were employed to evaluate the capability of TSMDA of predicting potential miRNA-disease associations in a real-world scenario.

The statistics reported in the 2020 annual report of the American Cancer Society show that these cancers are among the top five cancers with the highest estimated new cases and deaths in the US population.^40^ Breast cancer is widely known as the most prevalent cancer in females, accounting for 30% of the cases.^40^ Similarly, prostate cancer is the most commonly found male cancer, responsible for one-fifth of the cases, while lung cancer is the second most common type of cancer in both genders.^40^

In the first case study, the general predictive performance of TSMDA was assessed by its ability to identify the breast, prostate, and lung cancer-related miRNAs for experimentally validated associations in dbDEMC and miRCancer.^11^^,^^12^ Known associations in HMDD v.2.0 were chosen as a training dataset. The top 50 cancer-related miRNAs were ranked based on TSMDA scores and listed in Tables S2–S4. Using TSMDA scores, 49, 50, and 50 of the predicted miRNAs associated with breast, prostate, and lung cancer, respectively, were experimentally confirmed by other databases.

The ability of TSMDA to predict potential associations for diseases without verified associated miRNAs was evaluated in the second case study. Known associations between the three cancer types and miRNAs in the training set of HMDD v.2.0 were removed, one cancer at a time. As a result, 49, 49, and 49 of the top 50 were validated with known associations in dbDEMC and miR2Cancer (Tables S5–S7).^11^^,^^12^

In the third case study, miR2Disease containing 3,273 known associations between 349 miRNAs and 163 diseases was used to demonstrate our model performance on different datasets.^41^ miR2Disease was used to train the model, and the top 50 potential associated miRNAs predicted were investigated in dbDEMC and miR2Cancer (Tables S8–S10).^11^^,^^12^ All associations were confirmed, indicating the robustness of TSMDA to uncover potential miRNA-disease associations when considering different datasets.

### TSMDA web server

**Figure 3:**
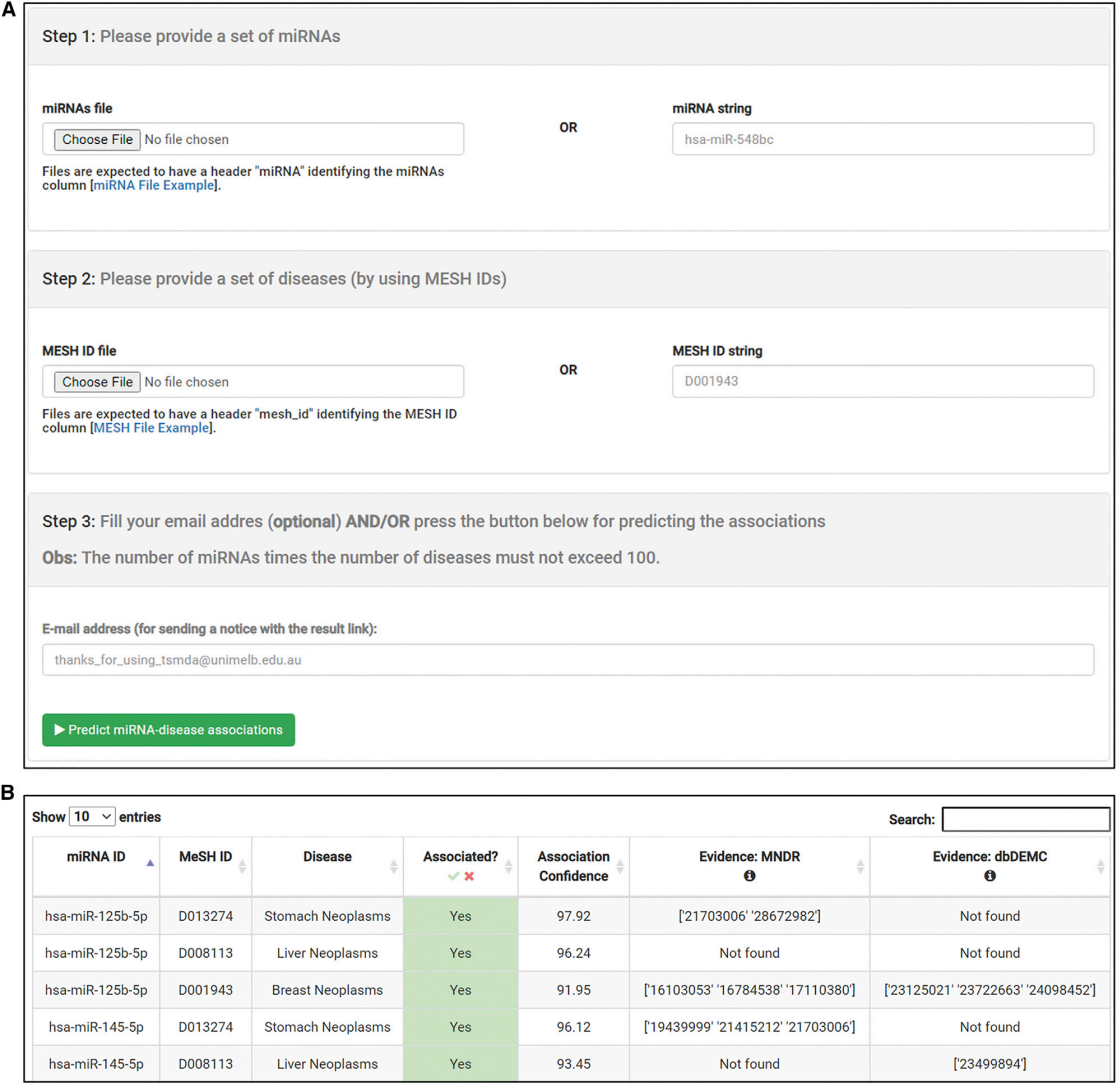
The TSMDA web server interface (A) A list of miRNAs in miRBase IDs and diseases in MeSH IDs are required as input for the TSMDA web server. (B) The result from TSMDA is provided as a table. A higher prediction score indicates a higher probability for miRNA-disease association. If a miRNA-disease association is experimentally supported by MNDR^31^ or dbDEMC,^11^ evidence is provided as a PMID.

We have made TSMDA available as an easy-to-use web server. the TSMDA web server works according to the following procedures. First, users are required to manually provide a list of miRNAs in miRBase format and a list of disease Medical Subject Heading (MeSH) IDs. This list can be provided as a file. Users also have the possibility to fill a single string for either miRNA or MeSH ID. The example can be downloaded in the TSMDA server (Figure 3A). After running TSMDA, prediction results will be provided as a table, which can be downloaded as a comma-separated file. For each pair of miRNA and disease, an association confidence is shown. A higher score indicates a higher potential of association between miRNA and disease. Moreover, related evidence is given as a PMID for a pair of miRNA and disease with existing experimental support in Mammalian ncRNA-Disease Repository (MNDR) or dbDEMC.^11^^,^^42^ The TSMDA web server is available at http://biosig.unimelb.edu.au/tsmda/.

## Discussion

The utilization of miRNAs as diagnostic biomarkers or drugs has received growing attention,^7^, ^8^, ^9^ due to their significant regulatory roles in various physiological processes. To enable the development of miRNA-based therapeutic applications, a wide range of studies has validated a large number of relationships between miRNAs and disease, which have provided a better understanding of miRNA regulatory mechanisms.^3^, ^4^, ^5^ A significant proportion of potential miRNA-disease associations are yet to be explored, and computational methods play an essential role in assisting on this task.

**Figure 4:**
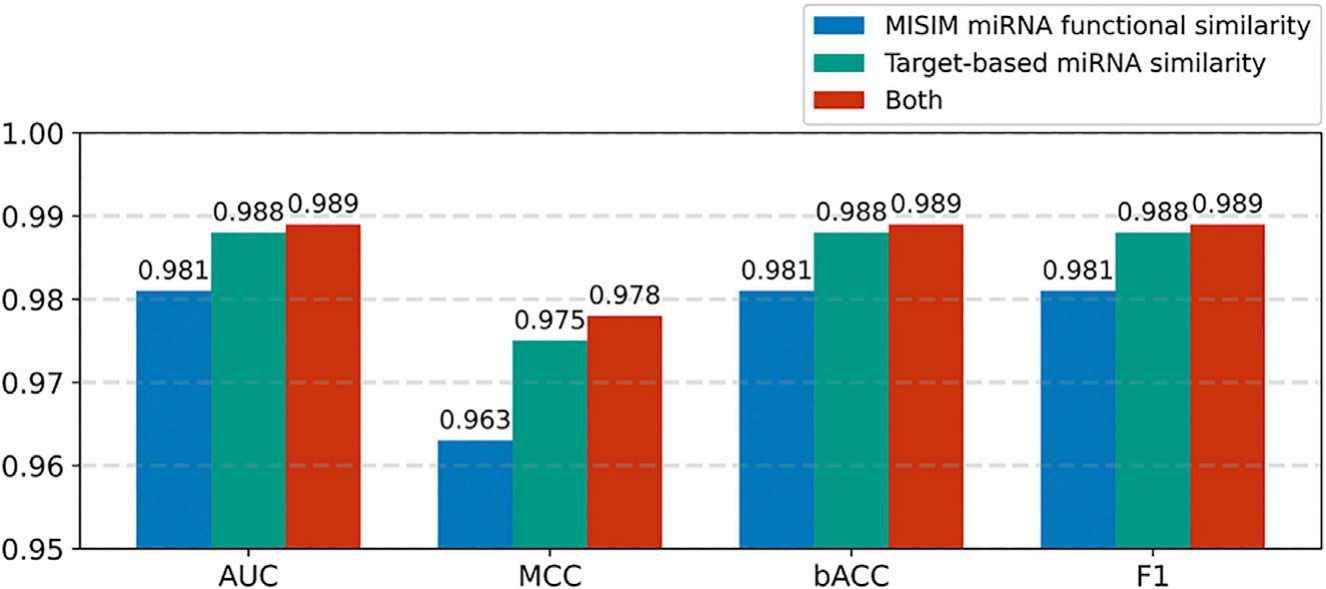
The introduction of miRNA functional similarity (MISIM) with target-based miRNA similarity moderately enhances TSMDA performance AUC, MCC, bACC, and F1 in TSMDA models with three sets of features—3 target-based miRNA similarities (T) with 5 symptom-based similarities, 5 MISIM similarities (M) with 5 symptom-based similarities, and 8 target-based and MISIM miRNA similarities (T + M) with 5 symptom-based similarities—were assessed in 5-fold cross-validation with XGBoost classifier.

The proposed TSMDA prediction model has led to three major improvements for miRNA-disease-association prediction in terms of (1) miRNA similarity calculation, (2) disease similarity calculation, and (3) negative sample selection strategies. First, an approach for miRNA similarity calculation called target-based miRNA similarity was introduced. Unlike sequence or associated-disease information used in many previous methods,^13^, ^14^, ^15^, ^16^, ^17^, ^18^, ^19^, ^20^, ^21^, ^22^ individual miRNAs’ target genes directly reflect their unique function in molecular pathways. TSDMA has shown that by combining this method with MISIM miRNA functional similarity, they can help improve the model’s prediction power and reliability (Figure 4). Second, the symptom-based approach was utilized to calculate disease similarity. Several studies indicated the remarkable predictive capability of symptom-based similarity as it is associated with several molecular mechanisms,^43^, ^44^, ^45^ including shared genes, protein interactions, and molecular origins. Finally, we designed modern negative sample selection approaches on TSMDA. A lack of actual negative samples has been a limitation of miRNA-disease-association studies for an extended period. In this work, two reliable methods proposed in previous research, miRNA-gene-disease network^18^ and traditional PU learning,^17^^,^^25^^,^^26^ were adopted and modified. A more comprehensive network was obtained in comparison with previous methods by integrating two datasets from miRTarbase and Tarbase.^46^^,^^47^ The modified PU learning approach was introduced to relieve the strong dependence on the chosen criteria of selecting reliable negative samples in the original method.^48^

To verify the performance of TSMDA, the method was assessed under different cross-validation schemes, as well as through an independent blind test and three case studies. The performance levels and consistency under different validation scenarios illustrate the robustness of the method in prioritizing potential miRNA-disease associations. Furthermore, we showed TSMDA has outperformed alternative state-of-the-art methods (Figure 2A),^14^^,^^17^^,^^19^, ^20^, ^21^, ^22^ indicating a substantial improvement from previous efforts. The model’s reliability in a real-world application was supported by the case studies on the three common cancer types. To facilitate access to the method’s capabilities and enable reproducibility, we developed a user-friendly web server to allow easy access by other researchers.

In future works, miRNA-disease-association predictions might be improved in many directions. One of the limitations of the current model is the bias in data availability. A significant proportion of experimentally validated miRNA-disease associations as well as miRNA-target gene interactions has not been confirmed. Although TSMDA has attempted to overcome this bias by introducing a unique weighting scheme, more informative data sources, such as miRNA expression profiles, should be taken into consideration. On the other hand, other molecular properties of diseases, such as related biochemical pathways, could be introduced to enhance predictive accuracy. However, the disease similarity estimation is restrained by the limitation of HMDD v.2.0, where some diseases are not found in the Disease Ontology,^49^ a standardized ontology for human diseases generally used for diverse disease similarity calculations.^50^^,^^51^

Data quality is a significant hurdle in determining the success of miRNA-disease-association prediction models. As future work, a practical method that utilizes other biological information to guide a reliable negative sample selection may be proposed to increase the model effectiveness. Furthermore, miRNA expression profiles retrieved from public databases, such as The Cancer Genome Atlas, can be utilized to improve data quality. Removing confirmed miRNA-disease associations with low confidence according to differential expression analysis may significantly improve data reliability.

## Materials and methods

### TSMDA general workflow

**Figure 5:**
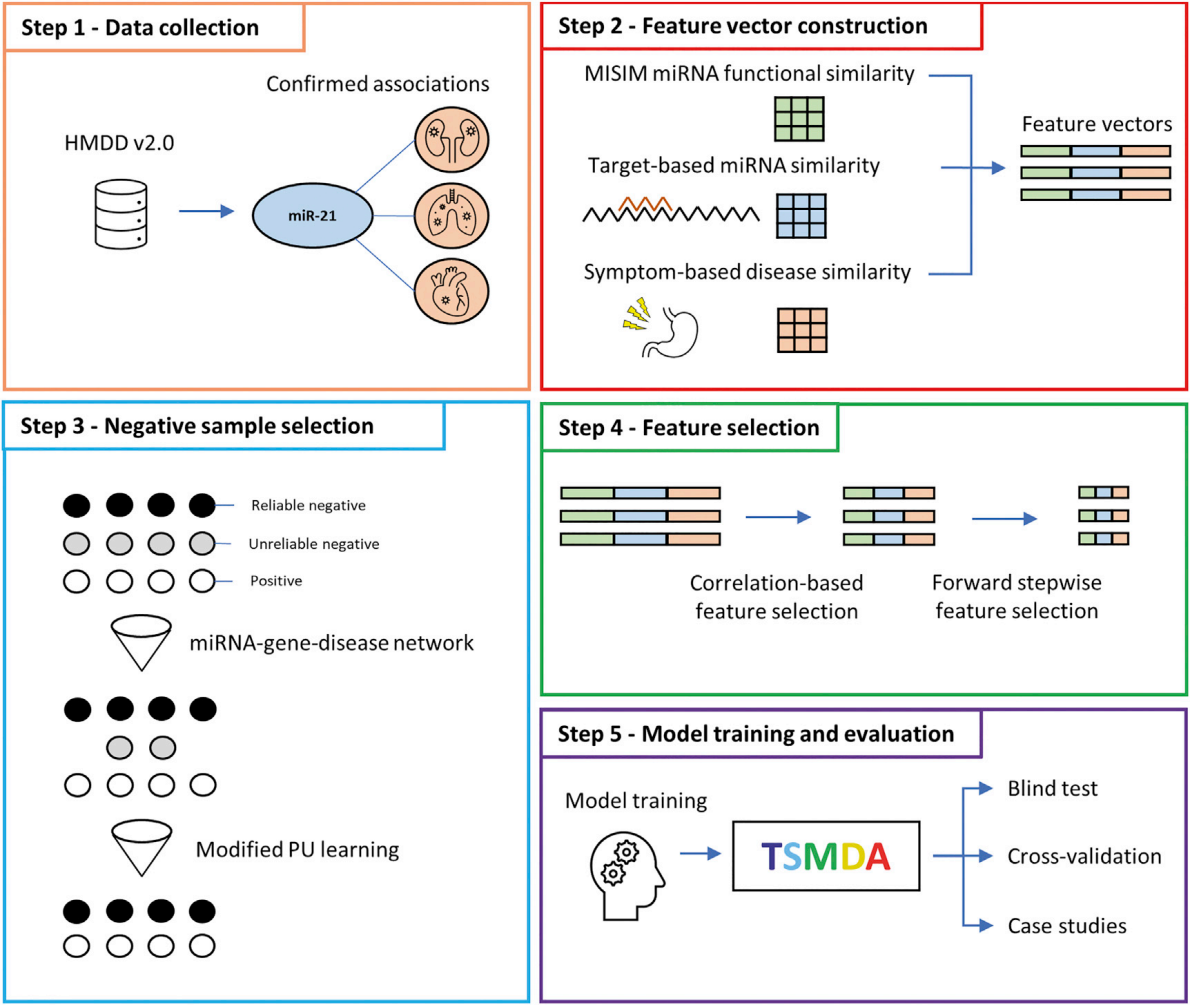
TSMDA: Predicting miRNA-disease associations The development of TSMDA is divided into five steps: (1) data collection, (2) feature vector construction, (3) negative sample selection, (4) feature selection, and (5) model training and evaluation.

The proposed pipeline consists of five main steps (Figure 5). First, confirmed miRNA-disease associations were obtained from HMDD v.2.0.^10^ In the following step, feature engineering is performed and three sets of similarities constructed: MISIM,^23^ target-based miRNA similarity, and symptom-based disease similarity. These were integrated into feature vectors, representing pairs of miRNA-disease associations. Subsequently, reliable negative samples were selected using miRNA-gene-disease network and modified PU learning. Following that, a subset of relevant features is chosen by correlation-based and forward stepwise greedy feature selection.^27^^,^^28^ An extreme gradient boosting classifier (XGBoost) was employed to create a prediction model for potential associations. The method’s performance was assessed using both internal (5-fold, 10-fold, and 20-fold cross-validation) and external validation (blind test and three case studies).^52^

### Data collection: Human miRNA-disease associations

Experimentally validated human miRNA-disease associations were retrieved from HMDD v.2.0.^10^ The dataset contains 5,430 associations between 495 miRNAs and 383 diseases. Given this dataset, a vector *V* was built to describe the associations between miRNA and disease as follows:

(Equation 1) $$\;V=\left(A_{i,j},A_{i+1,j},A_{i+2,j},\ldots ,A_{M\times D}\right),$$

where M and D are the number of miRNAs and diseases in HMDD v.2.0, respectively, and $A_{i,j}$ is equal to one (1) if miRNA *i* and disease *j* are experimentally associated, and zero (0), otherwise.

### miRNA functional similarity

The MISIM used in this research was proposed by Wang et al.^23^ due to its relative simplicity and decent capability to represent miRNA similarity in a number of studies.^14^, ^15^, ^16^, ^17^, ^18^, ^19^, ^20^, ^21^, ^22^ The data of known miRNA-disease associations was utilized to assess miRNA similarity based on the assumption that miRNAs with similar functions are more likely to be associated with pathologically similar diseases. We retrieved miRNA functional similarity of miRNAs found in HMDD v.2.0 from the Cui Lab repository. The miRNA functional similarity matrix (MFS) describing the pairwise similarities among 495 miRNAs was constructed.

### Target-based miRNA similarity

Despite a satisfactory contribution to miRNA-disease predictions, incomplete data of validated associations still limited the performance of MISIM. To address this limitation, other data types should be considered to enhance miRNA similarity representation and mitigate biases. Two modern methods, LFEMDA and DBMDA, proposed sequence-based approaches to estimate miRNA similarity. The improved accuracy indicated the usefulness of biological features.^15^^,^^16^

In this work, biological information of miRNA targets was introduced to determine miRNA similarity. miRNAs perform a regulatory function via complementary base pairing with several mRNAs. Thus, miRNAs with similar target genes are more likely to have similar functions in molecular pathways. Here, we utilized the numbers of shared target genes to assess miRNA similarity. The experimentally validated miRNA-target interactions were available at miRTarBase and TarBase.^46^^,^^47^ miRTarBase consists of 553,168 interactions between 3,775 miRNAs and 22,336 target genes, whereas TarBase contains 422,614 interactions between 1,084 miRNAs and 20,790 target genes. The interactions related to miRNAs found in HMDD v.2.0 were extracted and merged, producing the dataset of 397,402 interactions between 489 miRNAs and 21,284 genes. Across all 495 miRNAs in the HMDD v.2.0, six missing miRNAs were proved by miRBase to be experimental errors.^53^

The information of shared target genes between miRNAs was utilized to calculate miRNA similarity. The 21,284-dimensional vector M described target genes for miRNA i was created as:

(Equation 2) $$M_{i}=\left(s_{i,1},s_{i,2},s_{i,3},\ldots ,s_{i,j}\right),$$

where $s_{i,j}$ denotes the strength of the interaction between miRNA i and target gene j. It is calculated by taking the prevalence of target genes in the dataset into consideration. The strength of interaction between a pair of miRNA i and target gene j is equal to log 2 of term frequency of target gene if they are interacting, otherwise equal to zero as follows:

(Equation 3) $$s_{i,j}=\left\{ \begin{array}{ll} log_{2}F_{j} & M_{i}\;and\;T_{j}\;are\;interacting\\ 0 & otherwise \end{array}\right..$$

In the equation, $F_{j}$ is a term frequency of a target gene. $M_{i}$ and $T_{j}$ refer to miRNA i and target gene j.

In the end, cosine similarity was employed to assess the target-based miRNA similarity between the arrays representing the miRNAs.^54^ Cosine similarity is a standard metric used to compute the directional similarity between two vectors by capturing orientational differences. The advantage of the cosine similarity is the computation irrespective of vectors’ sizes. miRNA similarity was calculated as stored in a target-based miRNA similarity matrix (TMS).

### Symptom-based disease similarity

Several studies demonstrated a close correspondence between the resemblance of molecular pathogenesis (e.g., shared gene, protein-protein interactions, and molecular origin) and the phenotypic similarity in clinical symptoms.^55^^,^^56^ On this basis, Zhou et al.^43^ proposed the novel symptom-based disease similarity calculation that can be applied to create a phenotype network profile for discovering molecular targets for drug repurposing.^44^^,^^45^ This approach has displayed a robust correlation between calculated similarity and molecular-level disease components. The unique advantage of this method is a wide availability of directly observable clinical phenotypes in various diseases. For this reason, TSMDA aimed to implement a symptom-based approach to measure disease similarity.

The co-occurrences of diseases and symptoms in PubMed were used to characterize each disease in terms of clinical phenotypes. First, the 383 diseases from HMDD v.2.0 were mapped to 328 MeSH identifiers.^57^ For each disease, its MeSH ID was used as a query to search for co-occurrences with 481 symptoms (2020th updated), categorized by PubMed. Disease *i* can be described by a 481-dimensional vector as follows:

(Equation 4) $$D_{i}=\left(w_{i,1},w_{i,2},w_{i,3},\ldots ,w_{i,481}\right).$$

$w_{i,j}$ quantifies the intensity of the co-occurrence between disease i and symptom j. According to the bias where some symptoms such as pain are comparatively more abundant, the intensity was estimated considering the term frequency-inverse document frequency (TF-IDF).^43^ It is calculated from absolute co-occurrence $W_{i,j}$ as the following equation:

(Equation 5) $$w_{i.j}=W_{i,j\;}log\frac{N}{n_{j}},$$

where *N* denotes the number of diseases in HMDD v.2.0, while $n_{j}$ represents the number of diseases where symptom j appears. Same as target-based miRNA similarity, the cosine similarity was also employed to measure the directional similarity between symptom-described vectors for each disease.^54^ The symptom-based disease similarity among 495 diseases was represented as a symptom-based disease similarity matrix (SDS).

### miRNA and disease similarity integration

We obtained 1,373-dimensional feature vectors describing 189,585 possible pairs of miRNAs and diseases in HMDD v.2.0 from the integration of MISIM miRNA functional similarity, target-based miRNA similarity, and symptom-based disease similarity. The feature vectors $F_{i,j}$ representing miRNA i and disease j were constructed as follows:

(Equation 6) $$F_{i,j}=\left(mms_{i,1},\ldots ,mms_{i,nM},tms_{i,1},\ldots ,tms_{i,nM},sds_{j,1},\ldots ,sds_{j,nD}\right).$$

Here,$\;mms_{i,m}$ and $tms_{i.m}$ denote MISIM and target-based miRNA similarity between miRNAi and miRNAm, whereas $sds_{j,d}$ is the symptom-based disease similarity between disease jand disease d. nMand nD are numbers of miRNAs and diseases in HMDD v.2.0.

### Negative sample selection

Negative sample selection is undeniably one of the most crucial processes in miRNA-disease-association modeling due to the absence of true negative samples in the database. A variety of negative sample selection strategies have been explored to address this issue.

The general standard procedure is to obtain negative samples by a random selection from unlabeled miRNA-disease associations.^14^^,^^16^^,^^21^ This approach expects the ideal situation where unconfirmed pairs can be arbitrarily considered as not existing, which may not be valid, negatively affecting the reliability of negative samples. NSEMDA^17^ has proposed alternative strategies that utilize a traditional PU learning model^25^^,^^26^ to train the model and remove unreliable negative samples iteratively. In contrast, NMLPMDA suggested a distinct method that focused on the construction of a miRNA-gene-disease network.^18^ Pairs of miRNA and disease that show no relationship were selected as reliable negative samples. The remarkable accuracy of these methods illustrates the potential to prioritize reliable negative samples. However, there is still room for improvement.

TSMDA employed a miRNA-gene-disease network, followed by modified PU learning to form a robust negative sample selection. The methods were further improved by extending the size of the network and replacing the original PU learning with a modified algorithm. In details, 115,891,964 verified gene-disease associations between 21,671 genes and 30,170 diseases were acquired from DisGENET v.7.0.^58^ They were integrated with the aforementioned miRNA-target gene interactions from miRTarbase^46^ and Tarbase,^47^ forming the miRNA-gene-disease network. Pairs of miRNA and disease sharing the same gene in the network were considered as potential miRNA-disease associations. Unknown associations in our dataset were then mapped to the network to filter out the potential associations. From 184,155 unknown associations, only 20,716 associations (∼10%) are selected as promising negative samples.

To increasingly refine the negative samples, modified PU learning^48^ employing an iterative pruning strategy was introduced. It was initially proposed to mitigate the heavy dependence on the chosen criteria of reliable negative sample selection,^48^ resulting in more reliable negative samples. In this work, 20% of known associations in HMDD v.2.0 were separated from the dataset and used as positive samples in PU learning to prevent overfitting from a bias toward a dataset, while the remaining negative samples were negative samples. Random forest (RF) classifier^59^ was selected to train a model in an iterative manner because of the robustness to overfitting and less requirement for parameter tuning. Negative samples with low confidence scores were removed in each turn, otherwise retained in the dataset.

During the first loop, the RF classifier was trained to remove a large proportion of negative samples that were highly likely to be positive samples. Merely 1% of negative samples classified as positives or negatives, but with a probability lower than 95%, they were eliminated. Due to this strict condition, the remaining negative samples will be comparatively more reliable and suitable for training subsequent models. In the following loops, we aimed for a slight reduction of negative samples in each loop. An RF classifier was similarly implemented; however, the hyperparameter was set in order to limit the model complexity, allowing iterative pruning. The numbers of estimators and maximum depth were reduced to 20 and 3. Only negative samples classified as positives were removed each step. The process was run until the number of reliable samples was the same as known associations.

### Feature selection

After the negative sample selection, feature selection was used to define a better set of features, so redundancy and noise are removed or diminished, computation time and model complexity are reduced, and overfitting is less likely to happen.^52^ In several miRNA-disease-association models, employing a proper feature selection technique leads to a substantially increased predictive performance.^60^, ^61^, ^62^ TSMDA utilizes two feature selection means, a correlation-based^27^ and forward stepwise greedy feature selection.^28^^,^^63^, ^64^, ^65^

Initially, Pearson's correlation coefficients (PCCs) between every pair of features were calculated and represented as a heatmap in Figure S1. It was apparent that multiple features are redundant, so some can be discarded without reducing model accuracy. We conducted a performance evaluation to examine the optimal cutoff for PCC values (Figure S2). As a result, the cutoff of 0.6 was selected. If a PCC between features is higher than 0.6, only one feature is randomly retained. Consequently, the number of features was drastically reduced from 1,373 to 97.

Forward stepwise greedy feature selection was used to scale down the remaining dimensions by selecting the best combination of features.^28^ The process begins with zero features selected. The most useful feature contributing the most to the performance was included one at a time. In each step, 10-fold cross-validation with XGBoost^29^ was performed, then evaluated with MCC (Figure S3). At the end, 13 features (Table 1) were chosen as the best combination required to train a highly accurate model. The subset of features contained five miRNA functional similarities, three target-based miRNA similarities, and five symptom-based disease similarities.

### XGBoost classifier

XGBoost^29^ is one of the most widely used tree-based boosting algorithms, where a set of weak classifiers are combined to form a strong classifier sequentially. In each iteration, misclassification errors of a previous classifier were corrected to create a more accurate model. In contrast to other boosting algorithms, XGBoost has several enhancements in regularization, parallelization, handling missing values, dropout methods, and others.

In this work, this algorithm has been shown to be the one with best performances in terms of miRNA-disease-association predictions in preliminary experiments (see Table S11). The final feature vectors represented by the selected 13 features are adopted to train and validate the XGBoost classification model.

### Availability of data and materials

The datasets used in this work are available at http://biosig.unimelb.edu.au/tsmda/data.

## Supplemental information

Document S1. Tables S2–S11 and Figures S1–S3

Table S1. The contribution of each feature to a prediction based on SHAP values in misclassified entries in a blind test

Document S2. Article plus supplemental information
