# Supplementary material for: TSMDA: Target and symptom-based computational model for miRNA-disease-association prediction
Source: Mol Ther Nucleic Acids. 2021 Aug 26;26:536–46. doi: 10.1016/j.omtn.2021.08.016 (PMC8479276; doi:10.1016/j.omtn.2021.08.016)
Supplement: Document S1. Tables S2–S11 and Figures S1–S3 [file mmc1.pdf]

OMTN, Volume *26*

Supplemental information

TSMDA: Target and symptom-based computational model for miRNA-disease-association prediction

Korawich Uthayopas, Alex G.C. de Sá, Azadeh Alavi, Douglas E.V. Pires, and David B. Ascher

Supplemental Information

Supplementary Table S1. The contribution of each feature to a prediction based on SHAP values in misclassified entries in a blind test.

Supplementary Table S2. Top 50 potential breast neoplasms-associated miRNAs predicted by TSMDA based on known associations in HMDD v2.0. (1st column record top 1-25. 3rd column record top 26-50)

miRNA	Evidence	miRNA	Evidence
hsa-mir-32	dbDEMC; miRCancer	hsa-mir-33b	dbDEMC; miRCancer
hsa-mir-196b	dbDEMC	hsa-mir-200a	dbDEMC; miRCancer
hsa-mir-155	dbDEMC; miRCancer	hsa-mir-363	dbDEMC
hsa-mir-151a	dbDEMC	hsa-mir-150	dbDEMC; miRCancer
hsa-mir-320d	dbDEMC	hsa-mir-486	dbDEMC
hsa-mir-194	dbDEMC	hsa-mir-371a	dbDEMC
hsa-mir-31	dbDEMC; miRCancer	hsa-mir-429	dbDEMC; miRCancer
hsa-mir-376c	dbDEMC	hsa-mir-769	dbDEMC
hsa-mir-211	dbDEMC; miRCancer	hsa-mir-299	dbDEMC

hsa-mir-20a	dbDEMC; miRCancer	hsa-mir-370	dbDEMC; miRCancer
hsa-mir-224	dbDEMC; miRCancer	hsa-mir-381	dbDEMC; miRCancer
hsa-mir-451a	dbDEMC; miRCancer	hsa-mir-29a	dbDEMC; miRCancer
hsa-mir-491	dbDEMC	hsa-mir-507	unconfirmed
hsa-mir-337	dbDEMC	hsa-mir-206	dbDEMC; miRCancer
hsa-mir-874	dbDEMC; miRCancer	hsa-mir-624	dbDEMC
hsa-mir-199a	dbDEMC	hsa-mir-29b	dbDEMC
hsa-mir-181b	dbDEMC; miRCancer	hsa-mir-30c	dbDEMC; miRCancer
hsa-mir-320a	miRCancer	hsa-mir-483	dbDEMC; miRCancer
hsa-mir-382	dbDEMC	hsa-mir-342	dbDEMC
hsa-mir-21	dbDEMC; miRCancer	hsa-mir-223	dbDEMC; miRCancer
hsa-mir-93	dbDEMC; miRCancer	hsa-mir-25	dbDEMC; miRCancer
hsa-mir-16	dbDEMC; miRCancer	hsa-mir-30a	dbDEMC; miRCancer
hsa-mir-182	dbDEMC; miRCancer	hsa-mir-200b	dbDEMC; miRCancer
hsa-mir-205	dbDEMC; miRCancer	hsa-mir-298	dbDEMC
hsa-mir-17	dbDEMC; miRCancer	hsa-mir-147a	dbDEMC

Supplementary Table S3. Top 50 potential prostate neoplasms-associated miRNAs predicted by TSMDA based on known associations in HMDD v2.0. (1st column record top 1-25. 3rd column record top 26-50)

miRNA	Evidence	miRNA	Evidence
hsa-mir-211	dbDEMC; miRCancer	hsa-mir-223	dbDEMC; miRCancer
hsa-mir-32	dbDEMC	hsa-mir-423	dbDEMC
hsa-mir-151a	dbDEMC	hsa-mir-374b	dbDEMC; miRCancer
hsa-mir-31	dbDEMC; miRCancer	hsa-mir-507	dbDEMC
hsa-mir-155	dbDEMC; miRCancer	hsa-mir-25	dbDEMC; miRCancer
hsa-mir-196b	dbDEMC	hsa-mir-17	dbDEMC; miRCancer
hsa-mir-491	dbDEMC	hsa-mir-196a	dbDEMC
hsa-mir-20a	dbDEMC; miRCancer	hsa-mir-30c	dbDEMC; miRCancer
hsa-mir-182	dbDEMC; miRCancer	hsa-mir-342	dbDEMC
hsa-mir-370	dbDEMC	hsa-mir-221	dbDEMC; miRCancer
hsa-mir-320d	dbDEMC	hsa-mir-210	dbDEMC
hsa-mir-21	dbDEMC; miRCancer	hsa-mir-451a	dbDEMC
hsa-mir-194	dbDEMC; miRCancer	hsa-mir-146a	dbDEMC; miRCancer

hsa-mir-382	miRCancer	hsa-mir-331	dbDEMC
hsa-mir-376c	dbDEMC	hsa-mir-185	dbDEMC
hsa-mir-193b	dbDEMC; miRCancer	hsa-mir-339	dbDEMC
hsa-mir-205	dbDEMC; miRCancer	hsa-mir-200b	dbDEMC; miRCancer
hsa-mir-486	dbDEMC	hsa-mir-224	dbDEMC; miRCancer
hsa-mir-624	dbDEMC	hsa-mir-29a	dbDEMC
hsa-mir-381	dbDEMC	hsa-mir-15a	dbDEMC; miRCancer
hsa-mir-371a	dbDEMC	hsa-mir-363	dbDEMC
hsa-mir-93	dbDEMC; miRCancer	hsa-mir-199a	dbDEMC; miRCancer
hsa-mir-30d	dbDEMC; miRCancer	hsa-mir-206	dbDEMC; miRCancer
hsa-mir-33b	dbDEMC	hsa-mir-326	dbDEMC
hsa-mir-30e	dbDEMC; miRCancer	hsa-mir-26b	dbDEMC; miRCancer

Supplementary Table S4. Top 50 potential lung neoplasms-associated miRNAs predicted by TSMDA based on known associations in HMDD v2.0. (1st column record top 1-25. 3rd column record top 26-50)

miRNA	Evidence	miRNA	Evidence
hsa-mir-151a	dbDEMC	hsa-mir-184	dbDEMC
hsa-mir-32	dbDEMC; miRCancer	hsa-mir-451a	dbDEMC
hsa-mir-31	dbDEMC; miRCancer	hsa-mir-30e	dbDEMC
hsa-mir-211	dbDEMC	hsa-mir-30d	dbDEMC; miRCancer
hsa-mir-194	dbDEMC; miRCancer	hsa-mir-96	dbDEMC; miRCancer
hsa-mir-370	dbDEMC	hsa-mir-486	dbDEMC
hsa-mir-155	dbDEMC; miRCancer	hsa-mir-21	dbDEMC; miRCancer
hsa-mir-376c	dbDEMC	hsa-mir-34b	dbDEMC; miRCancer
hsa-mir-93	dbDEMC; miRCancer	hsa-mir-200a	dbDEMC; miRCancer
hsa-mir-382	dbDEMC	hsa-mir-374b	dbDEMC
hsa-mir-363	dbDEMC	hsa-mir-181b	dbDEMC; miRCancer
hsa-mir-451	dbDEMC	hsa-mir-483	dbDEMC
hsa-mir-185	dbDEMC	hsa-mir-17	dbDEMC; miRCancer

hsa-mir-381	dbDEMC	hsa-mir-205	dbDEMC; miRCancer
hsa-mir-371a	dbDEMC	hsa-mir-758	dbDEMC
hsa-mir-199a	dbDEMC	hsa-mir-298	dbDEMC
hsa-mir-423	dbDEMC	hsa-mir-30c	dbDEMC
hsa-mir-196b	dbDEMC; miRCancer	hsa-mir-208a	miRCancer
hsa-mir-320d	dbDEMC	hsa-mir-92	dbDEMC
hsa-mir-20a	dbDEMC; miRCancer	hsa-mir-193b	dbDEMC
hsa-mir-429	dbDEMC; miRCancer	hsa-mir-25	dbDEMC
hsa-mir-342	dbDEMC	hsa-mir-196a	dbDEMC; miRCancer
hsa-mir-491	dbDEMC	hsa-mir-182	dbDEMC; miRCancer
hsa-mir-339	dbDEMC	hsa-mir-16	dbDEMC
hsa-mir-507	dbDEMC	hsa-mir-206	dbDEMC; miRCancer

Supplementary Table S5. Top 50 potential breast neoplasms-associated miRNA predicted by TSMDA based on HMDD v2.0; however, the known associations related to breast neoplasms were deleted from the training dataset. (1st column record top 1-25. 3rd column record top 26-50)

**miRNA**	**Evidence**	**miRNA**	**Evidence**
hsa-mir-371a	dbDEMC	hsa-mir-211	dbDEMC; miRCancer
hsa-mir-181c	dbDEMC	hsa-mir-182	dbDEMC; miRCancer
hsa-mir-342	dbDEMC	hsa-mir-93	dbDEMC; miRCancer
hsa-mir-382	dbDEMC	hsa-mir-424	dbDEMC
hsa-mir-31	dbDEMC; miRCancer	hsa-mir-429	dbDEMC; miRCancer
hsa-mir-210	dbDEMC; miRCancer	hsa-mir-335	dbDEMC; miRCancer
hsa-mir-203	dbDEMC; miRCancer	hsa-mir-363	dbDEMC
hsa-mir-451a	dbDEMC; miRCancer	hsa-mir-151a	dbDEMC
hsa-mir-181b	dbDEMC; miRCancer	hsa-mir-185	dbDEMC; miRCancer
hsa-mir-370	dbDEMC; miRCancer	hsa-mir-485	dbDEMC
hsa-mir-206	dbDEMC; miRCancer	hsa-mir-451	dbDEMC; miRCancer
hsa-mir-323a	dbDEMC	hsa-mir-507	unconfirmed

hsa-mir-454	dbDEMC	hsa-mir-96	dbDEMC; miRCancer
hsa-mir-194	dbDEMC	hsa-mir-328	dbDEMC
hsa-mir-186	dbDEMC; miRCancer	hsa-mir-487b	dbDEMC
hsa-mir-769	dbDEMC	hsa-mir-663	dbDEMC
hsa-mir-17	dbDEMC; miRCancer	hsa-mir-340	dbDEMC; miRCancer
hsa-mir-381	dbDEMC; miRCancer	hsa-mir-483	dbDEMC; miRCancer
hsa-mir-216a	dbDEMC; miRCancer	hsa-mir-320a	miRCancer
hsa-mir-624	dbDEMC	hsa-mir-526b	dbDEMC; miRCancer
hsa-mir-32	dbDEMC; miRCancer	hsa-mir-193a	dbDEMC; miRCancer
hsa-mir-663b	dbDEMC	hsa-mir-196a	dbDEMC; miRCancer
hsa-mir-425	dbDEMC; miRCancer	hsa-mir-92	dbDEMC
hsa-mir-199a	dbDEMC	hsa-mir-330	dbDEMC
hsa-mir-181a	dbDEMC; miRCancer	hsa-mir-198	dbDEMC; miRCancer

Supplementary Table S6. Top 50 potential prostate neoplasms-associated miRNA predicted by TSMDA based on HMDD v2.0; however, the known associations related to breast neoplasms were deleted from the training dataset. (1st column record top 1-25. 3rd column record top 26-50)

**miRNA**	**Evidence**	**miRNA**	**Evidence**
hsa-mir-31	dbDEMC; miRCancer	hsa-mir-17	dbDEMC; miRCancer
hsa-mir-342	dbDEMC	hsa-mir-155	dbDEMC; miRCancer
hsa-mir-371a	dbDEMC	hsa-mir-29c	dbDEMC; miRCancer
hsa-mir-32	dbDEMC	hsa-mir-486	dbDEMC
hsa-mir-181c	dbDEMC; miRCancer	hsa-mir-192	dbDEMC; miRCancer
hsa-mir-370	dbDEMC	hsa-mir-18a	dbDEMC; miRCancer
hsa-mir-210	dbDEMC	hsa-mir-20a	dbDEMC; miRCancer
hsa-mir-181a	dbDEMC; miRCancer	hsa-mir-93	dbDEMC; miRCancer
hsa-mir-203	dbDEMC; miRCancer	hsa-mir-320a	unconfirmed
hsa-mir-451a	dbDEMC	hsa-mir-182	dbDEMC; miRCancer
hsa-mir-216a	dbDEMC	hsa-mir-184	dbDEMC
hsa-mir-186	dbDEMC; miRCancer	hsa-mir-507	dbDEMC

hsa-mir-206	dbDEMC; miRCancer	hsa-mir-30d	dbDEMC; miRCancer
hsa-mir-92	dbDEMC	hsa-mir-20b	dbDEMC; miRCancer
hsa-mir-429	miRCancer	hsa-mir-25	dbDEMC; miRCancer
hsa-mir-499a	dbDEMC	hsa-mir-454	dbDEMC; miRCancer
hsa-mir-211	dbDEMC; miRCancer	hsa-mir-34c	dbDEMC; miRCancer
hsa-mir-30e	dbDEMC; miRCancer	hsa-mir-223	dbDEMC; miRCancer
hsa-mir-214	dbDEMC	hsa-mir-423	dbDEMC
hsa-mir-487b	dbDEMC	hsa-mir-323a	dbDEMC
hsa-mir-382	miRCancer	hsa-mir-194	dbDEMC; miRCancer
hsa-mir-181b	dbDEMC; miRCancer	hsa-mir-185	dbDEMC
hsa-mir-451	dbDEMC	hsa-mir-21	dbDEMC; miRCancer
hsa-mir-200c	dbDEMC	hsa-mir-19b	dbDEMC
hsa-mir-30c	dbDEMC; miRCancer	hsa-mir-335	dbDEMC; miRCancer

Supplementary Table S7. Top 50 potential lung neoplasms-associated miRNA predicted by TSMDA based on HMDD v2.0; however, the known associations related to breast neoplasms were deleted from the training dataset. (1st column record top 1-25. 3rd column record top 26-50)

**miRNA**	**Evidence**	**miRNA**	**Evidence**
hsa-mir-342	dbDEMC	hsa-mir-196a	dbDEMC; miRCancer
hsa-mir-31	dbDEMC; miRCancer	hsa-mir-382	dbDEMC
hsa-mir-210	dbDEMC; miRCancer	hsa-mir-147a	dbDEMC
hsa-mir-371a	dbDEMC	hsa-mir-451	dbDEMC
hsa-mir-206	dbDEMC; miRCancer	hsa-mir-151a	dbDEMC
hsa-mir-769	dbDEMC	hsa-mir-485	dbDEMC
hsa-mir-381	dbDEMC	hsa-mir-199a	dbDEMC
hsa-mir-499a	dbDEMC	hsa-mir-181b	dbDEMC; miRCancer
hsa-mir-32	dbDEMC; miRCancer	hsa-mir-193a	dbDEMC
hsa-mir-451a	dbDEMC	hsa-mir-17	dbDEMC; miRCancer
hsa-mir-216a	dbDEMC	hsa-mir-425	dbDEMC
hsa-mir-203	dbDEMC; miRCancer	hsa-mir-520c	dbDEMC

hsa-mir-181c	dbDEMC	hsa-mir-185	dbDEMC
hsa-mir-663b	dbDEMC	hsa-mir-198	dbDEMC; miRCancer
hsa-mir-93	dbDEMC; miRCancer	hsa-mir-758	dbDEMC
hsa-mir-454	dbDEMC	hsa-mir-520d	dbDEMC
hsa-mir-323a	dbDEMC	hsa-mir-507	dbDEMC
hsa-mir-186	dbDEMC; miRCancer	hsa-mir-92	dbDEMC
hsa-mir-155	dbDEMC; miRCancer	hsa-mir-429	dbDEMC; miRCancer
hsa-mir-20a	dbDEMC; miRCancer	hsa-mir-500a	dbDEMC
hsa-mir-421	dbDEMC	hsa-mir-624	unconfirmed
hsa-mir-194	dbDEMC; miRCancer	hsa-mir-424	dbDEMC
hsa-mir-363	dbDEMC	hsa-mir-523	dbDEMC
hsa-mir-486	dbDEMC	hsa-mir-211	dbDEMC
hsa-mir-370	dbDEMC	hsa-mir-192	dbDEMC; miRCancer

Supplementary Table S8. Top 50 potential breast neoplasms-associated miRNAs predicted by TSMDA based on known associations in HMDD v2.0. (1st column record top 1-25. 3rd column record top 26-50)

miRNA	Evidence	miRNA	Evidence
hsa-miR-9-3p	dbDEMC	hsa-miR-25-3p	dbDEMC; miRCancer
hsa-miR-155-5p	dbDEMC; miRCancer	hsa-miR-29b-3p	dbDEMC
hsa-miR-224-5p	dbDEMC; miRCancer	hsa-miR-210-3p	dbDEMC; miRCancer
hsa-miR-17-3p	dbDEMC; miRCancer	hsa-miR-27a-3p	dbDEMC; miRCancer
hsa-miR-34a-5p	dbDEMC; miRCancer	hsa-miR-93-5p	dbDEMC; miRCancer
hsa-miR-15b-5p	dbDEMC	hsa-let-7a-5p	dbDEMC; miRCancer
hsa-miR-181a-5p	dbDEMC; miRCancer	hsa-miR-30b-5p	dbDEMC
hsa-miR-221-3p	dbDEMC; miRCancer	hsa-miR-222-3p	dbDEMC; miRCancer
hsa-let-7d-5p	dbDEMC; miRCancer	hsa-miR-223-3p	dbDEMC; miRCancer
hsa-miR-183-5p	dbDEMC; miRCancer	hsa-miR-19a-3p	dbDEMC; miRCancer
hsa-miR-106b-5p	dbDEMC; miRCancer	hsa-miR-197-3p	dbDEMC; miRCancer
hsa-miR-16-5p	dbDEMC; miRCancer	hsa-miR-145-5p	dbDEMC; miRCancer
hsa-miR-29c-3p	dbDEMC; miRCancer	hsa-let-7g-5p	dbDEMC; miRCancer

hsa-miR-96-5p	dbDEMC; miRCancer	hsa-miR-130a-3p	dbDEMC; miRCancer
hsa-miR-126-3p	dbDEMC; miRCancer	hsa-miR-141-3p	dbDEMC; miRCancer
hsa-miR-200b-3p	dbDEMC; miRCancer	hsa-miR-192-5p	dbDEMC
hsa-miR-24-3p	dbDEMC; miRCancer	hsa-miR-193b-3p	dbDEMC; miRCancer
hsa-miR-1-3p	dbDEMC; miRCancer	hsa-miR-101-3p	dbDEMC; miRCancer
hsa-miR-181b-5p	dbDEMC; miRCancer	hsa-miR-429	dbDEMC; miRCancer
hsa-miR-125b-5p	dbDEMC; miRCancer	hsa-let-7i-5p	dbDEMC; miRCancer
hsa-miR-214-3p	dbDEMC; miRCancer	hsa-miR-92a-3p	dbDEMC; miRCancer
hsa-miR-182-5p	dbDEMC; miRCancer	hsa-miR-29a-3p	dbDEMC; miRCancer
hsa-miR-195-5p	dbDEMC; miRCancer	hsa-miR-199a-5p	dbDEMC
hsa-miR-34c-5p	dbDEMC; miRCancer	hsa-miR-146b-5p	dbDEMC; miRCancer
hsa-let-7c-5p	dbDEMC; miRCancer	hsa-miR-335-5p	dbDEMC; miRCancer

Supplementary Table S9. Top 50 potential prostate neoplasms-associated miRNAs predicted by TSMDA based on known associations in HMDD v2.0. (1st column record top 1-25. 3rd column record top 26-50)

miRNA	Evidence	miRNA	Evidence
hsa-miR-17-3p	dbDEMC	hsa-miR-181b-5p	dbDEMC; miRCancer
hsa-miR-224-5p	dbDEMC; miRCancer	hsa-miR-197-3p	dbDEMC
hsa-miR-9-3p	dbDEMC; miRCancer	hsa-miR-27a-3p	dbDEMC; miRCancer
hsa-miR-155-5p	dbDEMC; miRCancer	hsa-miR-195-5p	dbDEMC; miRCancer
hsa-miR-34c-5p	dbDEMC; miRCancer	hsa-miR-182-5p	dbDEMC; miRCancer
hsa-miR-34a-5p	dbDEMC; miRCancer	hsa-miR-141-3p	dbDEMC; miRCancer
hsa-let-7d-5p	dbDEMC	hsa-miR-181a-5p	dbDEMC; miRCancer
hsa-miR-29c-3p	dbDEMC; miRCancer	hsa-let-7i-5p	dbDEMC
hsa-miR-1-3p	dbDEMC; miRCancer	hsa-miR-16-5p	dbDEMC; miRCancer
hsa-miR-15b-5p	dbDEMC; miRCancer	hsa-miR-200b-3p	dbDEMC; miRCancer
hsa-miR-210-3p	dbDEMC	hsa-miR-223-3p	dbDEMC; miRCancer
hsa-miR-214-3p	dbDEMC	hsa-miR-193b-3p	dbDEMC; miRCancer
hsa-miR-145-5p	dbDEMC; miRCancer	hsa-miR-125a-5p	dbDEMC

hsa-miR-106b-5p	dbDEMC	hsa-miR-146b-5p	dbDEMC; miRCancer
hsa-miR-96-5p	dbDEMC; miRCancer	hsa-miR-143-3p	dbDEMC; miRCancer
hsa-miR-130a-3p	dbDEMC; miRCancer	hsa-miR-429	miRCancer
hsa-miR-125b-5p	dbDEMC; miRCancer	hsa-miR-222-3p	dbDEMC; miRCancer
hsa-let-7c-5p	dbDEMC; miRCancer	hsa-miR-30a-3p	dbDEMC; miRCancer
hsa-miR-19a-3p	dbDEMC	hsa-miR-30a-5p	dbDEMC; miRCancer
hsa-miR-221-3p	dbDEMC; miRCancer	hsa-let-7g-5p	dbDEMC
hsa-miR-24-3p	dbDEMC; miRCancer	hsa-miR-34b-5p	dbDEMC; miRCancer
hsa-miR-183-5p	dbDEMC; miRCancer	hsa-miR-130b-3p	dbDEMC; miRCancer
hsa-miR-335-5p	dbDEMC; miRCancer	hsa-miR-20a-5p	dbDEMC; miRCancer
hsa-miR-17-5p	dbDEMC; miRCancer	hsa-miR-92a-3p	dbDEMC
hsa-miR-93-5p	dbDEMC; miRCancer	hsa-miR-196a-5p	dbDEMC

Supplementary Table S10. Top 50 potential lung neoplasms-associated miRNAs predicted by TSMDA based on known associations in HMDD v2.0. (1st column record top 1-25. 3rd column record top 26-50)

miRNA	Evidence	miRNA	Evidence
hsa-miR-34a-5p	dbDEMC; miRCancer	hsa-miR-126-3p	dbDEMC
hsa-miR-224-5p	dbDEMC	hsa-miR-24-3p	dbDEMC; miRCancer
hsa-miR-17-3p	dbDEMC	hsa-miR-181b-5p	dbDEMC; miRCancer
hsa-miR-29b-3p	dbDEMC; miRCancer	hsa-miR-195-5p	dbDEMC; miRCancer
hsa-miR-9-3p	dbDEMC; miRCancer	hsa-miR-106b-5p	dbDEMC
hsa-miR-210-3p	dbDEMC; miRCancer	hsa-miR-183-5p	dbDEMC
hsa-miR-125b-5p	dbDEMC; miRCancer	hsa-miR-1-3p	dbDEMC; miRCancer
hsa-miR-155-5p	dbDEMC; miRCancer	hsa-let-7a-5p	dbDEMC
hsa-miR-200b-3p	dbDEMC; miRCancer	hsa-miR-27a-3p	dbDEMC; miRCancer
hsa-miR-34c-5p	dbDEMC; miRCancer	hsa-miR-222-3p	dbDEMC
hsa-miR-221-3p	dbDEMC	hsa-miR-214-3p	dbDEMC; miRCancer
hsa-let-7c-5p	dbDEMC; miRCancer	hsa-miR-182-5p	dbDEMC; miRCancer
hsa-let-7d-5p	dbDEMC; miRCancer	hsa-miR-335-5p	dbDEMC

hsa-miR-197-3p	dbDEMC; miRCancer	hsa-miR-19a-3p	
hsa-miR-16-5p	dbDEMC	hsa-miR-192-5p	
hsa-miR-15b-5p	dbDEMC; miRCancer	hsa-miR-143-3p	dbDEMC; miRCancer
hsa-miR-146b-5p	dbDEMC	hsa-miR-145-5p	dbDEMC; miRCancer
hsa-miR-30b-5p	dbDEMC	hsa-miR-92a-3p	dbDEMC; miRCancer
hsa-miR-223-3p	dbDEMC; miRCancer	hsa-miR-122-5p	dbDEMC
hsa-miR-93-5p	dbDEMC; miRCancer	hsa-miR-130a-3p	dbDEMC
hsa-miR-193b-3p	dbDEMC	hsa-miR-141-3p	dbDEMC; miRCancer
hsa-miR-25-3p	dbDEMC	hsa-let-7g-5p	dbDEMC; miRCancer
hsa-miR-96-5p	dbDEMC; miRCancer	hsa-miR-429	dbDEMC; miRCancer
hsa-miR-181a-5p	dbDEMC	hsa-miR-22-3p	dbDEMC; miRCancer
hsa-miR-29c-3p	dbDEMC; miRCancer	hsa-miR-302a-3p	dbDEMC

Supplementary Table S11. The performances of TSMDA under different classifiers in HMDD v2.0.

Algorithms	Area Under the Curve	Matthew's Correlation Coefficient	Balanced Accuracy	F1
XGBoost	0.988 ± 0.000	0.977 ± 0.000	0.988 ± 0.000	0.988 ± 0.000
Gradient Boosting	0.986 ± 0.001	0.974 ± 0.001	0.986 ± 0.001	0.986 ± 0.001
ADABoost	0.986 ± 0.000	0.973 ± 0.001	0.986 ± 0.000	0.986 ± 0.000
Random Forest	0.985 ± 0.000	0.970 ± 0.000	0.985 ± 0.000	0.985 ± 0.000
Extra Trees	0.978 ± 0.001	0.957 ± 0.001	0.978 ± 0.001	0.978 ± 0.001
Neural network	0.977 ± 0.001	0.954 ± 0.001	0.977 ± 0.001	0.977 ± 0.001
J48	0.969 ± 0.001	0.937 ± 0.001	0.969 ± 0.001	0.968 ± 0.001
SVC	0.953 ± 0.000	0.905 ± 0.000	0.953 ± 0.000	0.953 ± 0.000
KNN	0.944 ± 0.001	0.888 ± 0.002	0.944 ± 0.001	0.945 ± 0.001
Gaussian	0.937 ± 0.001	0.874 ± 0.001	0.938 ± 0.001	0.937 ± 0.001

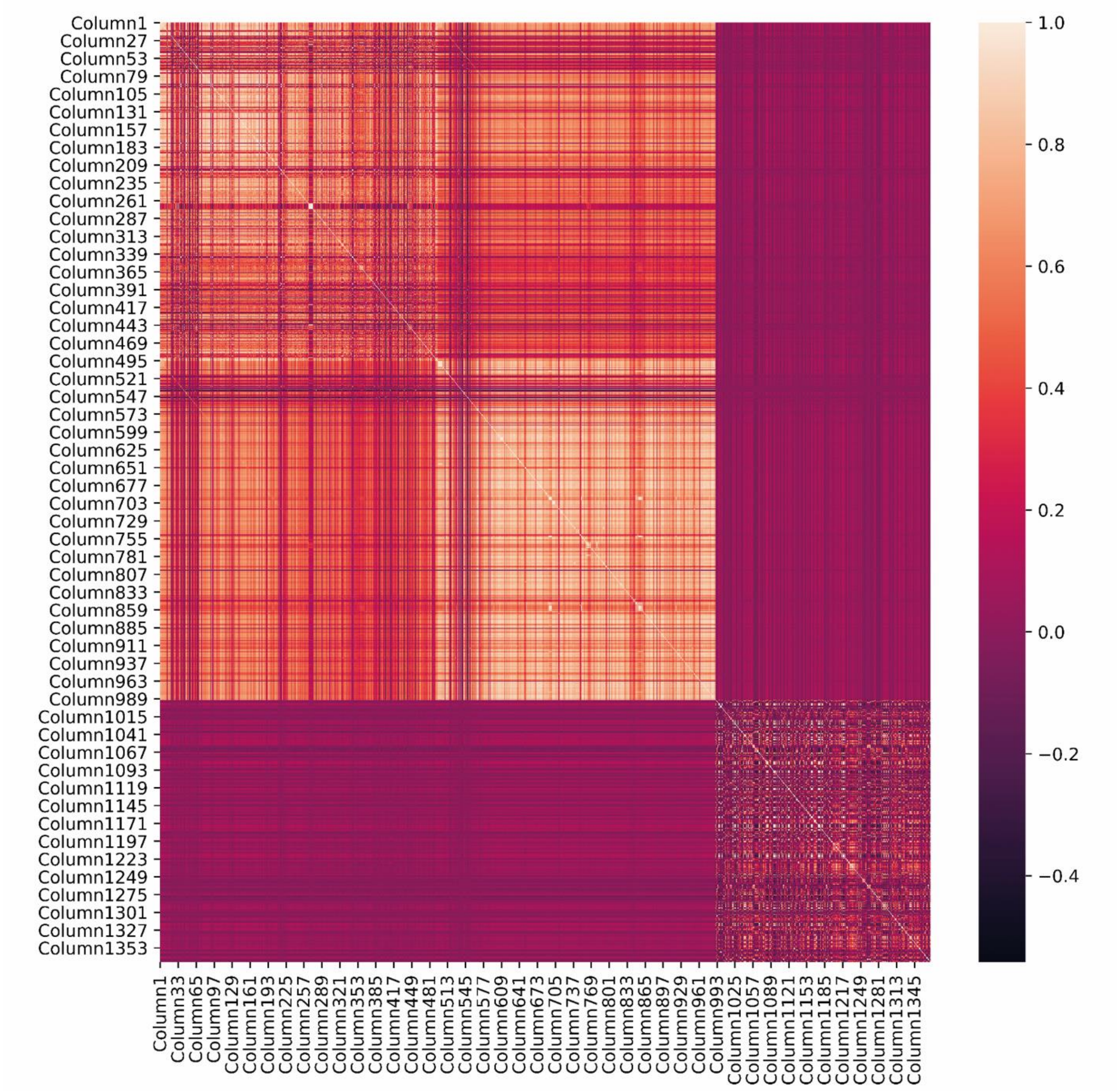

Supplementary Figure S1. The high levels of Pearson correlation coefficients indicate the massive redundancies between 1373 original features. A heat-map showing a correlation for every feature was constructed. Feature 1 – 495 account for MISIM miRNA functional similarity. Feature 495 – 990 account for target-based miRNA similarity. Feature 991 – 1373 account for symptom-based disease similarity**.**

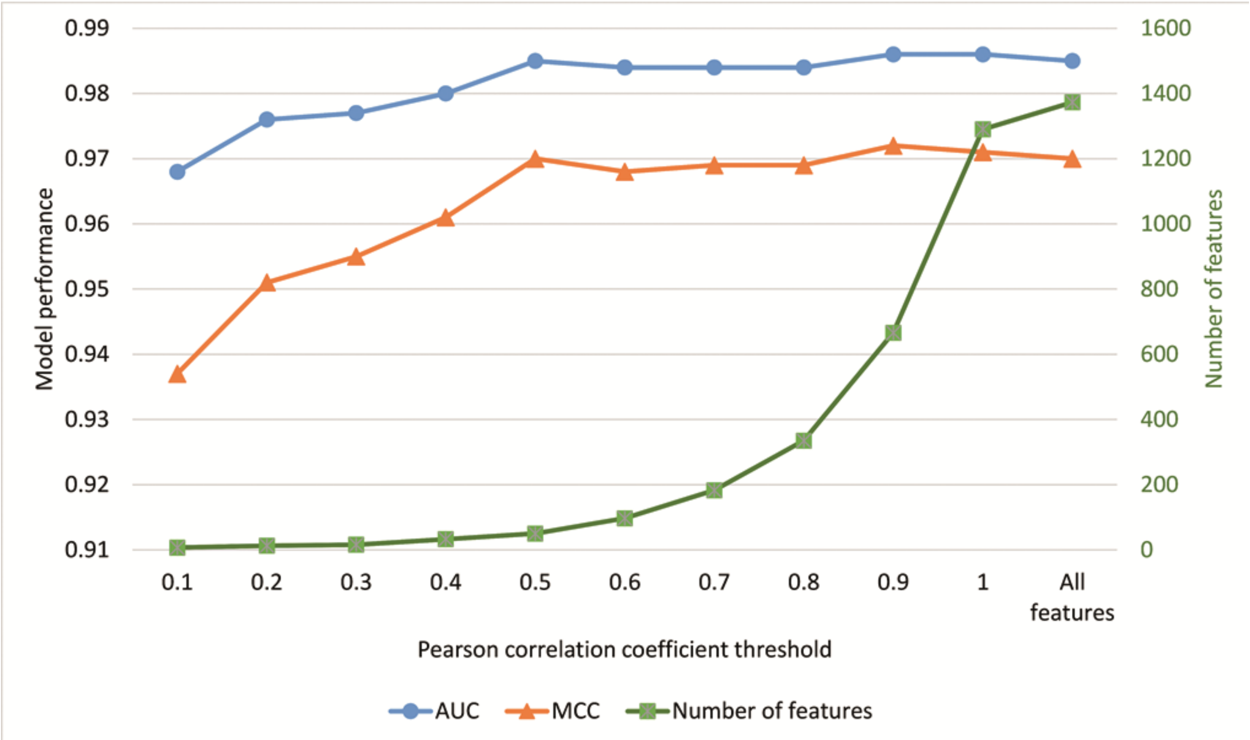

Supplementary Figure S2. Thresholds of Pearson correlation coefficient (PCC) between 0.5 and 0.7 are a reasonable cutoff in Correlation-based feature selection. Sets of features selected with correlation-based feature selection in thresholds between 0.1 and 1.0 were tested to investigate the optimal cutoff. If a PCC between features is higher than a threshold, only one feature is randomly retained. The number of features increases as the threshold is higher. Area Under the Curve and Matthew's Correlation Coefficient on 5-fold cross-validation with an extreme gradient boosting algorithms are assessed in each threshold.

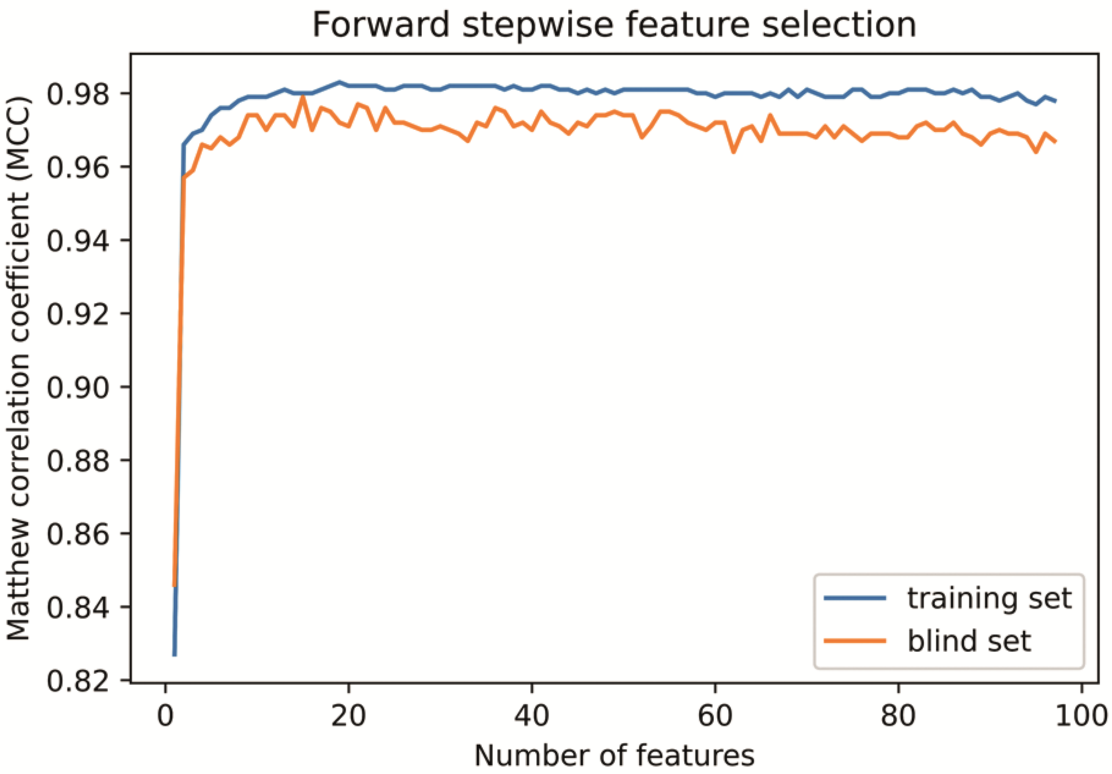

Supplementary Figure S3. 13 features - 5 miRNA functional similarities (MISIM), 3 Target-based miRNA similarities, and 5 Symptom-based disease similarities - are the effective, minimal combination required to train a highly accurate model. Forward stepwise feature selection was implemented to reduce the dimensions by selecting the best combination of features. The process begins with a zero feature selected, then the most useful feature to the performance was included one at a time. In each step, Matthew's correlation coefficient on 10-fold cross-validation with an extreme gradient boosting classifier was evaluated. A dataset was divided into a training and blind set to reduce overfitting problem.
